# Analysis of Age-Related Circular RNA Expression Profiles in Mesenchymal Stem Cells of Rat Bone Marrow

**DOI:** 10.3389/fgene.2021.600632

**Published:** 2021-06-28

**Authors:** Hui Sun, Yanan Sun, Xiao Yu, Xingyu Gao, Huan Wang, Lin Zhang, Yingai Shi, Xu He

**Affiliations:** The Key Laboratory of Pathobiology, Ministry of Education, College of Basic Medical Sciences, Jilin University, Changchun, China

**Keywords:** circular RNA, bioinformatics, senescence, MSCs, high-throughput sequencing

## Abstract

As multicellular organisms age, they undergo a reduction in tissue and organ function. Researchers have put forward a theory that stem cell aging is the main factor responsible for decreased tissue and organ function. The adult stem cells guarantee the maintenance and repair of adult tissues and organs. Among adult stem cells, mesenchymal stem cells (MSCs) are emerging as hopeful candidates for cell-based therapy of numerous diseases. In recent years, high-throughput sequencing technologies have evolved to identify circular RNAs (circRNAs) associated with an increasing number of diseases, such as cancer and age-related diseases. It has been reported that circRNAs can compete with microRNAs (miRNAs) to affect the stability or translation of target RNAs and further regulate gene expression at the transcriptional level. However, the role of circRNAs expressed in MSCs in aging mechanisms has not yet been deciphered. The aim of this study was to explore and analyze the expression profiles of age-related circRNAs in MSCs. In this study, bone marrow MSCs were extracted from aged and young rats and analyzed using high-throughput sequencing and bioinformatics. The reliability of high-throughput RNA sequencing was verified by quantitative real-time polymerase chain reaction. The most important circRNA functions and pathways were further selected by Gene Ontology (GO) and Kyoto Encyclopedia of Genes and Genomics (KEGG) analysis. Age-related circRNAs were found in the circrNA–miRNA–mRNA interaction network. The results of high-throughput sequencing showed that 4,229 circRNAs were involved in age-related senescence of MSCs. Compared with the young group, there were 29 differentially expressed circRNAs in the aged group, of which four were upregulated and 25 were downregulated. GO analysis covered three domains: biological process (BP), cellular component (CC), and molecular function (MF). The terms assigned to the BP domain were cellular metabolic processes and cellular macromolecule metabolic processes. The identified CC terms were intracellular and intracellular part, and the identified MF terms were binding and protein binding. The top five KEGG pathways were mitophagy–animal–*Rattus norvegicus*, prostate cancer–*Rattus norvegicus*, pathways in cancer–*Rattus norvegicus*, lysosome–*Rattus norvegicus*, and autophagy–animal–*Rattus norvegicus*. Altogether, circRNAs may play a major role in age-related MSC senescence. This study provides new mechanistic insights into MSC senescence, possibly leading to novel therapeutic strategies for age-related diseases.

## Introduction

Currently, with the improvement in medical and health conditions, the average life span of human beings is gradually increasing, but this is also accompanied by a series of social problems, such as the aging of the population. Aging is one of the important reasons for population aging (Martineau and Plard, 2018). Aging is considered to be an inevitable change in the genome at different levels of cells and organisms, which can manifest as an accumulation of DNA damage, imbalance in protein homeostasis, changes in cell communication, and stem cell failure (Lopez-Otin et al., 2013). Aging is a major underlying factor in the high incidence of diseases, such as cancer, cardiovascular diseases, lung diseases, diabetes, and neurological diseases (Sameri et al., 2020). The process of cell growth is dynamic and systematic. The body’s organs and tissues can replenish and maintain optimal working conditions of the whole organism *via* the processes of cell replication, growth, and death (Jung and Brack, 2014). Adult stem cells are involved in these processes in many different tissues, and they are essential for tissue homeostasis and repair after injury. Tissue homeostasis and regenerative capacity rely on rare populations of somatic stem cells with the potential to self-renew and differentiate (Keyes and Fuchs, 2018).

At present, MSCs have become the main seed cells in stem cell tissue engineering. In order to obtain sufficient bone marrow mesenchymal stem cells for clinical treatment, it is necessary to expand the cells (Khademi-Shirvan et al., 2020). However, MSCs can also undergo senescence with the culture *in vitro* (Beane et al., 2014). Many researchers investigated the effect of MSC senescence on differentiation potential, immunomodulation ability, and migratory ability (Duggal and Brinchmann, 2011; Ma et al., 2017). MSCs are capable of self-renewal and multi-lineage differentiation into various tissues. MSCs exhibited decreased differentiation potential and the imbalanced differentiation with senescence. Some reports indicated that the osteogenic ability of MSCs deteriorated progressively as a function of increasing lifespan (Banfi et al., 2000; Cheng et al., 2011). Long-term cultured rat MSCs *in vitro* displayed a great differentiation potential impairment, with full loss of osteogenic potential and diminished adipogenic potential (Geissler et al., 2012). The immunomodulatory activity of senescent MSC was also altered, because senescent cells secrete a myriad of factors, including growth factors, proteases, and cytokines, with potent autocrine and paracrine activities. For example, IL-8 induces chemotaxis in neutrophils and other granulocytes (Baggiolini and Clark-Lewis, 1992). Sepulveda et al. found that radiation-induced senescence abrogated MSC-protective immunoregulatory effects in a mouse model of sepsis (Sepulveda et al., 2014). Altered immunomodulation ability of MSCs was associated with migratory defect and functional engraftment (Sepulveda et al., 2014). Transcriptome analysis shows that in MSCs which have been long-term cultured, genes regulating some chemokines, cytokines, and their receptors, cell migration are downregulated. MSCs lost their migratory and homing ability during *in vitro* culture (Geissler et al., 2012). During aging, MSCs undergo some detrimental alterations, such as changes in the microenvironment, a decline in the regenerative capacity, and loss of function, and the deviation of the forms of differentiated cells, which could drive the deterioration in tissue function and diminished capacity for tissue repair in older individuals. Therefore, MSCs play a vital role in preventing the aging of organs and tissues, and can delay aging. It is crucial to convert senescent stem cells into youthful stem cells *via* regulation to improve age-related phenotypes and treat age-related diseases.

Circular RNA (circRNA) is a new type of endogenous non-coding RNA (Patop et al., 2019) and has become a recent research hotspot in the field of molecular biology. CircRNAs are mainly produced by the sequence of exons or introns and require reverse complementary sequences or RNA-binding proteins (RBPs) for their biogenesis (Li et al., 2018). CircRNA expression is cell specific and increases during cell maturation (Haddad and Lorenzen, 2019). Unlike traditional linear RNAs, circRNAs are characterized by closed-loop structures without 5–3 polarity or a polyadenylated tail. Their annular structure gives them strong resistance to RNA exonucleases or RNase R (Lasda and Parker, 2014), and they often exhibit tissue/developmental stage-specific expression. The functional circRNAs were first found to act as microRNA sponges in 2013 (Aird and Zhang, 2014). Only a few years later, additional functions of circRNAs were discovered. It has been recently revealed that circRNAs can function as microRNA (miRNA) sponges, regulators of splicing and transcription, and modifiers of parental gene expression (Qu et al., 2015). CircRNA biogenesis, regulation, and expression patterns are widely implicated in multiple aspects of biology and diseases, including cancer, heart diseases, synaptic transmission, and aging (Qu et al., 2017, 2018; Yang et al., 2018).

Recent studies have found that circRNAs regulate cellular senescence and cell survival and may be regulators of aging and age-related diseases (Beerman and Rossi, 2014). Dysfunction of alternative splice forms during aging may lead to increased neurologic circRNA biosynthesis (Costantino et al., 2016). It has been reported that circFoxo3 is significantly expressed in myocardial samples from elderly patients as well as mice, suggesting that circFoxo3 may be a hallmark of cellular senescence (Du et al., 2017). One of the important features of skin photoaging is the reduction in type I collagen synthesis. Twenty-nine circRNAs with significantly different expressions were identified in human dermal fibroblasts by RNA-seq, and further experiments showed that circCOL3A1-859267 regulated the synthesis of type I collagen (Zhang et al., 2016). The expression of genes involved in aging photoreceptors in response to stress and DNA damage was increased, leading to decreased gene expression required for neuronal function. In contrast, circRNA levels were noticeably enhanced with age (Hariton et al., 2018).

Among the above studies, circRNAs act as functional sponges of miRNAs and proteins, and transcriptional regulatory factors of the host gene provide new insights into the study of aging and age-related diseases. Therefore, circRNAs are expected to become new biomarkers and targets for aging and age-related diseases (Zhang et al., 2018). However, there are a few studies on the regulatory role of circRNAs in the aging process of mesenchymal stem cells (MSCs). In this study, we used high-throughput RNA sequencing to explore the differential expression profiles of circRNAs in bone marrow MSCs obtained from aged and young rats during physiological aging. Age-related circRNAs were then comprehensively analyzed.

## Materials and Methods

### MSC Isolation and Culture

Male Wistar rats were obtained from the Experimental Animal Center of Jilin University, Changchun, China (permit number: SYXK 2018-0001). Primary MSCs were isolated from the bone marrow of young (1–2-month-old) and old (15–18-month-old) male Wistar rats using the whole bone marrow adherent method (Ma et al., 2017). First, a 10 ml syringe was used to wash the marrow cavity, and MSCs were aseptically isolated from the femurs. MSCs were then harvested and plated into 10 cm culture dishes in complete medium containing 89% Dulbecco’s modified Eagle medium with nutrient mixture F-12 (Gibco; Life Technologies, Grand Island, NY, United States) supplemented with 10% fetal bovine serum (Gibco; Life Technologies, Grand Island, NY, United States) and 1% penicillin streptomycin (TransGen Biotech; Beijing, China). After incubation for 24 h at 37°C with 5% CO_2_, non-adherent cells were removed and the culture medium was replaced every 3 days. When the cells reached approximately 80% confluency, MSCs were digested using 0.25% trypsin-EDTA (Gibco; Life Technologies, Grand Island, NY, United States) and passaged at a 1:3 ratio.

### Detection of Senescence-Associated Changes in MSCs

MSCs termed as young MSCs and old MSCs were obtained from young and aged rats, respectively. Cellular senescence was evaluated by observing cellular morphology using a phase-contrast microscope, detecting the activity of senescence-associated β-galactosidase (SA-β-gal) and mRNA expression of senescence-related factor P16^*INK*4*A*^ and P21^*WAF*1/CIP1^. According to the manufacturer’s instructions, SA-β-gal staining was performed by using a senescence cell histochemical staining kit (Beyotime, Shanghai, China). The primers for P16^*INK*4*A*^ and P21^*WAF*1/CIP1^ are listed in Table 1.

**TABLE 1:** Primers used in RT-qPCR.

Gene	Forward primer (5′–3′)	Reverse primer (5′–3′)	Variation tendency
β-Actin	GGAGATTACTGCCCTGGCTCCTA	GACTCATCGTACTCCTGCTTGCTG	
P21 ^*WAF1/CIP1*^	GACATCACCAGGATCGGACAT	GCAACGCTACTACGCAAGTAG	Upregulated
P16^ INK4A^	AACACTTTCGGTCGTACCC	GTCCTCGCAGTTCGAATC	Upregulated
Circ-cd2ap	TCAGGAGGAATCAGAGACAA	GAGGGAAACAGTCCCAACTT	Upregulated
Circ-Phldb2	AGAGGGAGCAAGATCACTTC	GCTTTACCTTCTCCTCTTTC	Upregulated
Circ-Mdm2	TGAGAAGCAGCAGCACATTG	GTTTTGGTCTAACCTTGTTG	Downregulated
Circ-Rab31	GCTCTTTCAGGGGATCATTC	GAATCCTGCTTAGTGATGTC	Downregulated

### High-Throughput Sequencing of circRNAs and Differential Expression Analysis

We used TRIzol reagent to extract total RNA from each sample according to the protocol (Trakunram et al., 2019). The quantity and quality of RNA were measured using a NanoDrop ND-1000 spectrophotometer (NanoDrop Technologies, Inc., Wilmington, DE, United States). The Agilent 2100 Bioanalyzer evaluates RNA characteristics by standard denaturing agarose gel electrophoresis. Then, using the spectrophotometer, 260 and 280 nm wavelengths were selected to measure total RNA, and we chose a sample with a 260/280 absorbance ratio of approximately 2.0. Kangcheng Biological Co., Ltd. (Changchun, China); conducted a quantitative analysis of circRNA; and built a library after removing ribosomal RNA for high-throughput RNA sequencing. Arraystar was used to identify circRNAs. Differentially expressed circRNAs were detected using the CIRCexplorer2. Two criteria were chosen: (I) | log2(foldchange)| > 1.5 and (II) *P*-value (*P* < 0.05).

### Construction of the circRNA–miRNA–mRNA Interaction Network

Based on increasing evidence, it is speculated that circRNAs may regulate the activity of miRNAs by acting as competing endogenous RNAs or miRNA sponges. A circRNA–miRNA–mRNA co-expression network was built to instruct the role of circRNAs in MSCs undergoing senescence. We used the top one upregulated circRNA and two downregulated circRNAs that we were interested in to construct this network using Cytoscape software version 3.7.1. Putative interactions between miRNAs and circRNAs were evaluated using miRanda (3.3a). Some miRNAs with differential expression in high-throughput sequencing results were chosen, and we used mirdbV6 to predict the mRNAs of these miRNAs. Target scores >90 were selected to build the circRNA–miRNA–mRNA interaction network.

### Gene Ontology (GO) and Kyoto Encyclopedia of Genes and Genomes (KEGG) Pathway Analysis

Gene ontology analysis describes the attributes of genes and gene products of many organisms through functional categories and cellular locations. We used DAVID to analyze the potential functions of mRNAs. *P*-values < 0.05 were considered statistically significant. GO can be divided into three parts: biological process (BP), cellular component (CC), and molecular function (MF). The five most enriched GO terms were ranked by *P* value. We performed the KEGG pathway analysis to determine the different biological pathways involved in mRNA, this analysis also used DAVID.

### Protein–Protein Interaction Network Analysis

The mRNAs were analyzed using the online tool STRING^1^. High confidence (interaction score > 0.7) was selected. The protein–protein interaction (PPI) network was drawn using Cytoscape software version 3.7.1.

### Analysis of the Expression Levels of mRNAs and circRNAs Using RT-qPCR

Gene expression levels were determined using real-time quantitative polymerase chain reaction (RT-qPCR) with TransStart Top Green qPCR SuperMix (TRANS, China) in a 7300 Real-Time PCR System (ABI, Vernon, CA, United States). The reliability of high-throughput RNA sequencing has been further verified. The top two upregulated circRNAs and two downregulated circRNAs of interest were chosen. SYBR Green Supermix was used for RT-qPCR. The expression was determined using the threshold cycle (Ct). β-Actin was used as the standard internal control, and each reaction was performed in triplicate.

## Results

### Age-Associated Changes in MSCs

**FIGURE 1:**
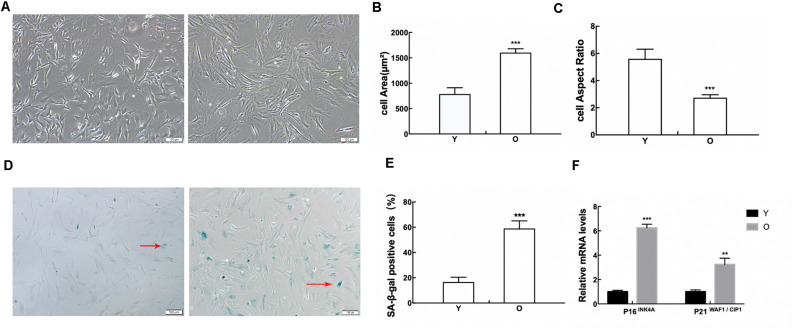
Detection of senescence-associated changes in MSCs. **(A)** Morphological alterations were observed under a phase-contrast microscope. The cell areas of MSCs in the old group were significantly increased **(B)**, and the cell aspect ratios were clearly reduced **(C)**. **(D–E)** SA-β-gal staining. As indicated by the red arrows, senescent cells were stained blue. The ratio of SA-β-gal-positive cells was much higher in MSCs obtained from old rats than those obtained from young rats. **(F)** RT-qPCR analyses of mRNA expression of the senescence-related factor pl6^*INK*4*A*^ and p21^*WAF*1/CIP1^. MSCs obtained from old rats expressed elevated levels of pl6^*INK*4*A*^ and p21^*WAF*1/CIP1^. Data indicate the mean ± *SD*, *n* = 3. **P < 0.01, ***P < 0.001 vs. Young (Y).

MSCs in the old group appeared flattened and enlarged, lost their stereoscopic perception, and contained clearly visible particles in the cytoplasm. While the cell borders of MSCs in the young group were distinct, the cells were long and fusiform in shape (Figure 1A). The analytic results showed that the cell areas of the MSCs obtained from old rats progressively increased (Figure 1B), while the cell aspect ratios gradually decreased (Figure 1C). The results of staining for SA-β-gal indicated that the ratio of SA-β-gal-positive cells was much higher in the MSCs obtained from old rats than those obtained from young rats (Figures 1D,E). We further monitored the mRNA expression of classical senescence markers to evaluate cell senescence at the molecular level, including pl6^*INK*4*A*^ and p21^*WAF*1/CIP1^, using RT-qPCR. As shown in Figure 1F, both pl6^*INK*4*A*^ and p21^*WAF*1/CIP1^ are upregulated in the MSCs obtained from old rats. These data displayed that MSCs obtained from old rats presented senescent alterations.

### Expression Patterns of circRNAs in Age-Associated MSC Senescence

**FIGURE 2:**
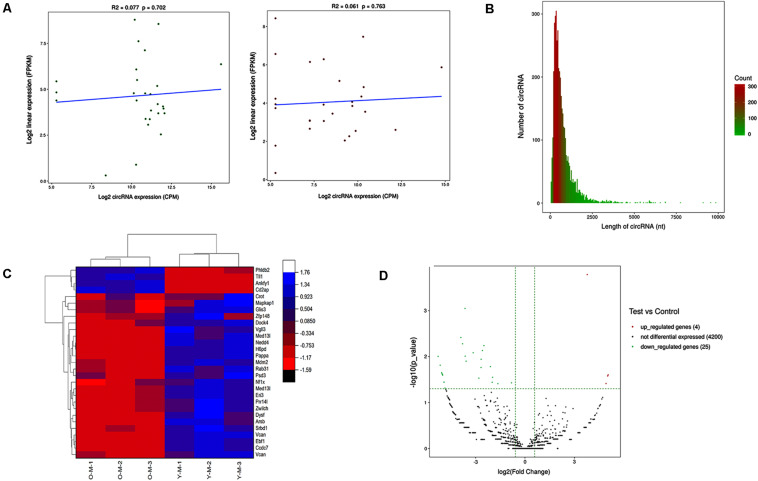
Results of high-throughput sequencing. **(A)** Expression correlation test of inter-sample. Young and old MSCs derived from 1–2- to 15–18-month-old rats were used, and three samples were contained in each group. The relativity between gene expression levels in the three samples indicated biological repetition. **(B)** CircRNA length distribution. It was predicted that approximately 95% of circRNAs had limited length of less than 2,000 nt. **(C)** Heatmap showing differential expression profiles of circRNAs between the two study groups and the homogeneity within each group. **(D)** Volcano map of differentially expressed circRNAs. The green and red dots in the figure represent the differentially expressed circRNAs, which are statistically significant. The upregulated circRNAs are represented by red dots, and the green dots denote the downregulated circRNAs.

The expression profiles of circRNAs were confirmed by high-throughput sequencing. Including young and old MSCs, each group contained three samples. The correlation between gene expression levels in different samples indicated biological repetition (Figure 2A). The sequencing data analysis indicated 4,229 differently expressed circRNAs between young and old MSCs. The length of these circRNAs ranged from 42 to 9,873 nucleotides (nt). Approximately 95% of circRNAs were predicted to be less than 2,000 nt (Figure 2B). Among the 4,229 circRNAs, four were markedly upregulated and 25 were clearly downregulated. A total of 4,200 circRNAs were not significantly different. The heatmap indicates the gene expression patterns of the samples (Figure 2C). Red is upregulated, and blue is downregulated.

### Identification of Differentially Expressed circRNAs

The | log2 (foldchange)| > 1.5 and *P*-value (*P* < 0.05) were used to evaluate significant differences in the expression of circRNAs between the two groups. Compared with the control group, there were 29 circRNAs significantly differentially expressed in the experimental group. Among them, the volcano plot showed that the expression of four circRNAs was upregulated and 25 were downregulated (Figure 2D).

### Validation of circRNA Expression

**FIGURE 3:**
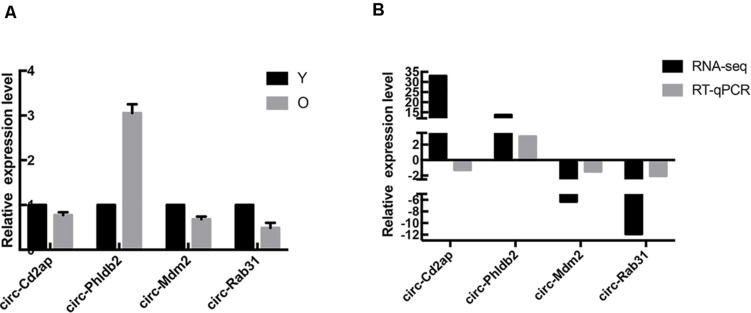
RT-qPCR results. **(A)** RT-qPCR detected the expression of the selected circRNAs. **(B)** The three expression patterns of the four circRNAs are consistent with the trends of high-throughput sequencing analysis results.

In order to verify the results of high-throughput sequencing, the top two upregulated and two downregulated circRNAs were selected, and their expression levels were measured by RT-qPCR analysis; results are shown in Figure 3A. Compared with the control group, the expression of Circ-Phldb2 was significantly upregulated in the experimental group, while the expressions of circ-Mdm2, circ-Rab31, and Circ-Cd2ap were downregulated in the experimental group. The expression patterns of these circRNAs were consistent with the trend of high-throughput sequencing analysis. However, the expression of Circ-Cd2ap conflicted with the high-throughput sequencing results (Figure 3B). The sequences of the primers used in RT-qPCR are shown in Table 1.

### Construction of the circRNA–miRNA–mRNA Interaction Network

**FIGURE 4:**
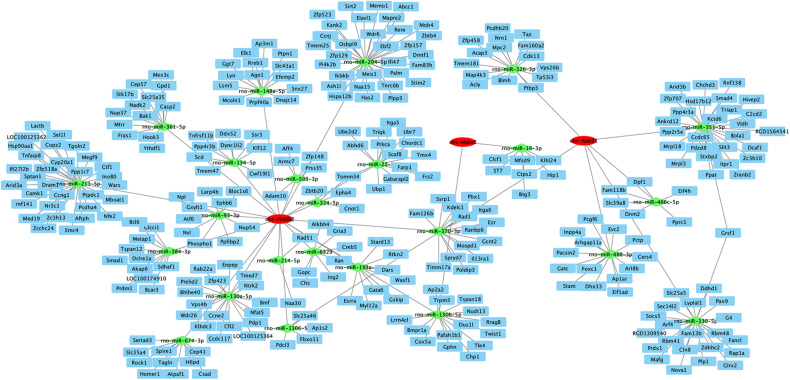
Network of CircRNA–miRNA–mRNA interaction. Cytoscape was used to construct the network of two downregulated and one upregulated circRNA. Putative interactions between miRNAs and circRNAs were predicted using mirTarbase 7.0. We used mirdbV6 to predict the miRNAs’ target genes. Target scores >90 were selected. In this figure, rectangles represent mRNAs, ovals represent circRNAs, and triangles represent miRNAs.

We constructed a co-expression network of circRNA–miRNA–mRNA to assess the potential functions of circRNAs and drew the circRNA–miRNA–mRNA interaction network using Cytoscape (Figure 4). Interestingly, circRNAs may play a central role in this network, and a single circRNA may be associated with many miRNAs, so as to affect many mRNAs. This network may contribute to clarifying the potential functions of circRNAs and their related mechanisms.

### GO and KEGG Analysis of mRNA

**FIGURE 5:**
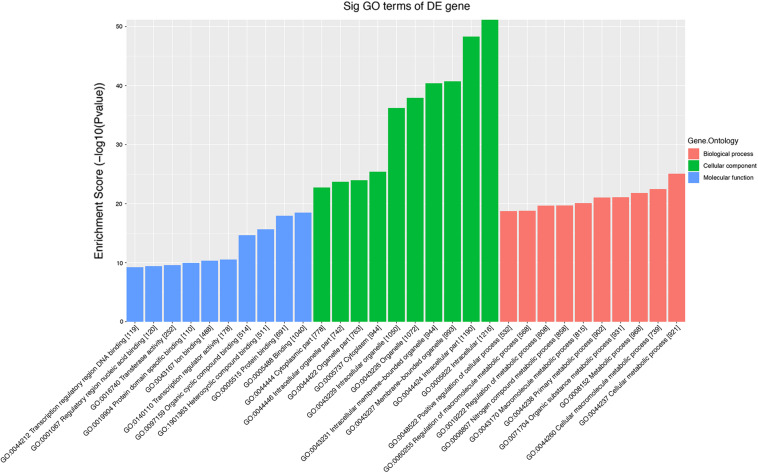
Top 10 GO terms from BP, CC, and MF. The top 10 GO terms in each group were ranked by *P*-value. The top five items are cellular metabolic process, cellular macromolecule metabolic process, metabolic process, organic substance metabolic process, and primary metabolic process (from BP). Intracellular, intracellular part, membrane-bounded organelle, intracellular membrane-bounded organelle, and binding (from CC). Binding, protein binding, heterocyclic compound binding, organic cyclic compound binding, and transcription regulator activity (from MF).

**FIGURE 6:**
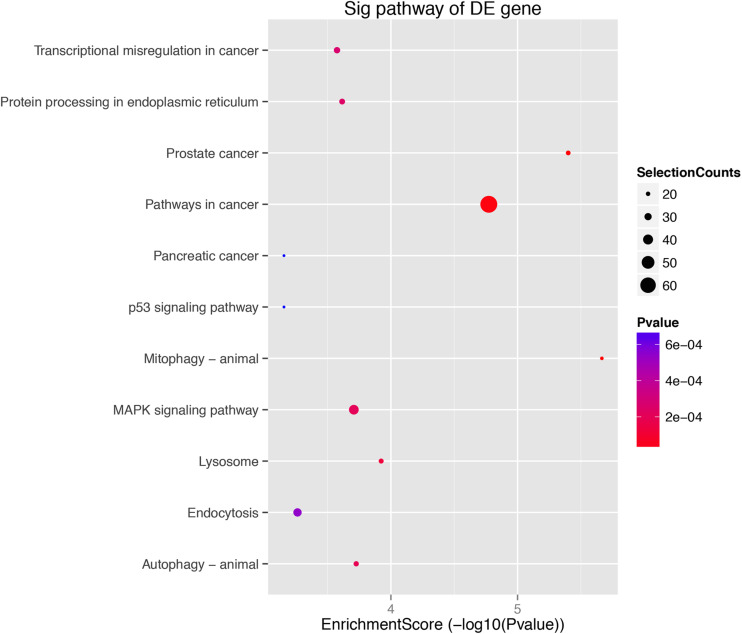
KEGG analysis. KEGG pathway analysis was used to determine the involvement of mRNAs in different biological pathways. The size of each circle indicates the number of circRNAs. The color of the circle indicates the *P*-value. The larger the circle and the lower the *P*-value, indicating a richer and more meaningful path.

The role of circRNAs in age-related MSC senescence was investigated by performing a GO functional analysis. The mRNAs from the circRNA–miRNA–mRNA interaction network were used in this analysis. GO has three different domains: BP, CC, and MF. The top (ranked by *P* value) BP terms, CC terms, and MF terms are shown in Figure 5 and Table 2. The identified BP terms were cellular metabolic process, cellular macromolecule metabolic process, metabolic process, organic substance metabolic process, and primary metabolic process. The identified CC terms were intracellular, membrane-bounded organelles, intracellular membrane-bound organelles, and binding. The identified MF terms were binding, protein binding, heterocyclic compound binding, organic cyclic compound binding, and transcription regulator activity. The top five (ranked by *P* value) KEGG pathways are shown in Figure 6 and Table 3. The identified pathways were mitophagy, prostate cancer, pathways in cancer, lysosomes, and autophagy. Notably, BCL2L1, E2F1, HRAS, MAPK8, MITF, RELA, and SP1 were found in both mitophagy and cancer pathways.

**TABLE 2:** The top five (ranked by *P*-value) BP terms, CC terms, and MF terms.

GO	*P*-value	Description	GO terms
GO:0044237	8.30345E-26	Cellular metabolic process	Biological_process
GO:0044260	3.30351E-23	Cellular macromolecule metabolic process	Biological_process
GO:0008152	1.52209E-22	Metabolic process	Biological_process
GO:0071704	8.02965E-22	Organic substance metabolic process	Biological_process
GO:0044238	8.89488E-22	Primary metabolic process	Biological_process
GO:0005622	6.90829E-52	Intracellular	Cellular_component
GO:0044424	4.84279E-49	Intracellular part	Cellular_component
GO:0043227	1.83709E-41	Membrane-bounded organelle	Cellular_component
GO:0043231	3.94388E-41	Intracellular membrane-bounded organelle	Cellular_component
GO:0043226	1.16141E-38	Organelle	Cellular_component
GO:0005488	3.22418E-19	Binding	Molecular_function
GO:0005515	1.08989E-18	Protein binding	Molecular_function
GO:1901363	2.17285E-16	Heterocyclic compound binding	Molecular_function
GO:0097159	2.16347E-15	Organic cyclic compound binding	Molecular_function
GO:0140110	2.87275E-11	Transcription regulator activity	Molecular_function

**TABLE 3:** The top five (ranked by *P*-value) KEGG pathways.

Term	Count	*P*-value	Genes
Mitophagy–animal—Rattus. norvegicus (rat)	17	2.16011E-06	BCL2L1//E2F1//FUNDC1//GABARAPL2//HRAS//MAPK8// MITF//NBR1//OPTN//RAB7A//RAB7B//RELA//RH OT1//RHOT2//SP1//SQSTM1//USP15
Prostate cancer. Rattus.Norvegicus. (rat)	21	3.98163E-06	BCL2//CCND1//CCNE2//CDK2//CDKN1B//CREB5//CREB BP//E2F1//ETV5//FGFR2//HRAS//HSP90AA1//IGF1//IKBK B//MAPK1//PDGFRA//PLAT//RELA//SOS1//TCF7L2//ZEB1
Pathways in cancer—Rattus. norvegicus (rat)	65	1.6866E-05	ABL1//ADCY6//APAF1//ARNT2//BAK1//BCL2//BCL2L1//B CL2L11//BDKRB2//CCND1//CCNE2//CDK2//CDKN1B//C KS2//CREBBP//DVL1//E2F1//EDN1//EDNRA//ELK1//ET S1//FGFR2//FZD1//GNA12//GNB4//GNG3//HES1//HRAS// HSP90AA1//IGF1//IKBKB//IL13RA1//IL7//ITGA6//JAG1//L PAR1//LRP6//MAPK1//MAPK8//MGST1//MITF//NFE2L2// NFKB2//PDGFRA//PIM1//PLCB4//PRKCA//PTGER2//RAD 51//RASGRP3//RELA//ROCK1//RPS6KA5//RUNX1T1//SK P2//SMAD2//SMAD4//SOS1//SP1//STAT3//TCF7L2//TFG// TRAF3//VEGFA//WNT10B
Lysosome–Rattus norvegicus. (rat)	22	0.000119359	AP1B1//AP1S1//AP1S2//AP1S3//AP3B1//AP3M1//AP4 S1//CLN5//CLTC//CTSL//GGA1//GGA2//GUSB//IDUA//IGF 2R//LAMP1//LIPA//M6PR//MCOLN1//NEU1//PPT2// SLC17A5
Autophagy–animal—Rattus. norvegicus (rat)	23	0.00018791	ATG16L1//BCL2//BCL2L1//CTSL//GABARAPL2//HRAS//I TPR1//LAMP1//MAPK1//MAPK8//MTMR3//PPP2CB//RAB 7A//RAB7B//RB1CC1//RGD1359108//RRAGB//RRAGC//S NAP29//SQSTM1//STX17//ULK2//ZFYVE1

### PPI Network

**FIGURE 7:**
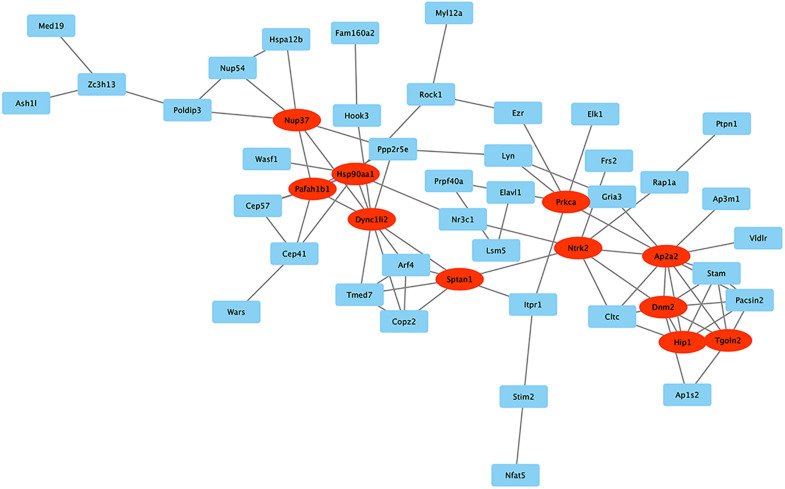
PPI network. STRING was used to predict protein interactions among the mRNA. The circRNAs with interaction scores >0.7 (high confidence) were chosen to construct PPI networks. Proteins with >5 relationships with other proteins are represented by ovals and may be the hub proteins in this network.

STRING was used to predict protein interactions between mRNAs. Interaction scores higher than 0.7 (high confidence) were selected for constructing PPI networks (Figure 7). Eleven proteins, Nup37, Hsp90aa1, Pafah1b1, Dync1li2, Sptan1, Prkca, Ntrk2, Ap2a2, Dnm2, Hip1, and Tgoln2, were strongly correlated with other proteins (connected with > 5 proteins). These hub proteins may play crucial roles in MSC senescence.

## Discussion

Our understanding of the role of circRNAs has increased due to the development of high-throughput sequencing technology. In recent years, some studies have found that circular RNA, as a member of the non-coding RNA family, plays important roles in different species. CircRNAs play vital roles in human diseases, such as age-related diseases (Cai et al., 2019). The functions and biological characteristics of circRNAs may help clarify the mechanisms underlying these diseases. The senescence of adult stem cells restricts their clinical applications, and the related mechanisms remain poorly understood. In this study, high-throughput sequencing was used to analyze the circRNA expression profiles of young and old MSCs, and it was further explored whether differentially expressed circRNAs may be involved in the age-related process of senescence of MSCs.

In the present study, MSCs were extracted from 1–2 to 15–18-month-old rats. MSCs obtained from the aged group changed significantly in morphology, such as increased cell areas, unclear boundary, and decreased aspect ratio, when compared with the young group. The positive ratio of SA-β-gal staining was elevated, and the senescence-associated factors p16^*INK*4*A*^ and p21^*WAF*1/CIP1^ were also obviously enhanced.

High-throughput RNA sequencing identified 4,299 differentially expressed circular RNAs in the two groups. A total of 29 circRNAs were significantly differentially expressed [*P* < 0.05, log2(foldchange) > 1.5]. The top one upregulated and two downregulated circRNAs validated by RT-qPCR were significantly dysregulated; this result is consistent with the high-throughput sequencing results, indicating the high reliability of high-throughput sequencing data. The expression of the top one upregulated and two downregulated circRNAs was confirmed by RT-qPCR, and the results were the same as the high-throughput sequencing results, confirming the reliability of the high-throughput sequencing data results. To further explore the regulatory role of circRNAs in age-related MSC senescence, a circRNA–miRNA–mRNA interaction network was constructed. The network suggested that circRNAs may play a vital regulatory role. As shown in the figure, one circRNA may be related to many downstream miRNAs, which in turn can regulate more downstream mRNAs. For example, in the results of this study, circ-Phldb2 may bind to rno-miR-130a-5p, rno-miR-134-5p, or rno-miR-208a-5p. These miRNAs may be related to a variety of mRNAs, including Ces2h, Tmem47, and Nova1. Decreased Nova1 in the aged brain might be due to general neuronal aging, as neurons are subjected to increased oxidative stress, perturbed energy homeostasis, DNA damage, and accumulation of misfolded/aggregated proteins (Beane et al., 2014).

KEGG and GO analyses were performed to determine the differentially expressed mRNAs in the two groups of MSCs, and the potential regulatory role of circRNAs in the senescence of MSCs was further studied. The BPs, CCs, and MFs of mRNA are annotated with GO analysis results. The analysis results indicate that the highly expressed mRNA may be related to cellular metabolic processes and cellular macromolecule metabolic processes. Abnormal metabolism of macromolecules can manifest as loss of protein stability, failure to repair DNA damage, and abnormal mRNA processing. Genome homeostasis is lost during aging, and telomeres at the ends of chromosomes can maintain genome stability. Some researchers have found that the telomere length of subcutaneous adipose tissue and leukocytes of obese individuals decreases (Duggal and Brinchmann, 2011). Loss of proteostasis has also been described as an important feature of aging. It has been reported that apart from the well-recognized role as a structural support, the age-related ECM protein also affects many important cell processes within the body. For example, the most abundant in the extracellular matrix is collagen and elastin and their changes can disrupt many different aspects of homeostasis and health functions (Birch, 2018). Studies have found that compared with young mice, aged mice have increased endoplasmic reticulum stress markers and decreased autophagy in adipose tissue (Ghosh et al., 2017).

KEGG analysis showed that mitochondrial autophagy was the most significantly associated pathway. This classical pathway is involved in many cellular metabolic processes, consistent with the results from the GO analysis. KEGG analysis showed that the pathway was the most abundant in cancer, with 65 related genes. Cancer cells have been shown to promote growth, survival, proliferation, and long-term maintenance by reprogramming their metabolism (Yu et al., 2019). Most ATP in tumor cells is synthesized through glycolysis, which is similar to stem cells and is inhibited during aging. Surprisingly, BCL2L1, E2F1, HRAS, MAPK8, MITF, RELA, and SP1 were all found in the mitophagy and cancer pathways. Therefore, further experiments are needed to show that these genes may play a role in the cross talk between different signaling pathways. At present, there are more researches on the relationship between E2F1 and circular RNA in tumors. E2F1 has an additional cyclin-binding domain and it can mediate both cell proliferation and p53-dependent/independent apoptosis. Researchers revealed that circCAMSAP1 might function as the sponge of miR-328-5p to regulate the expression of transcription factor E2F1, thereby promoting colorectal cancer (CRC) progression (Zhou et al., 2020). The suppression of the E2F1 pathway could be prevented by circSDHC binding competitively to miR-127-3p, thereby leading to renal cell carcinoma (RCC) malignant progression (Cen et al., 2021). Regarding the relationship between E2F1 and stem cells, researchers found that E2F1 supports the proliferation of neural stem cells (NSC) and that the E2F1-miRNA feedback loop is an important regulator of NSC fate determination. Therefore, E2F1 may also interact with age-related circular RNAs in MSCs to regulate cell proliferation and senescence (Palm et al., 2013). Increasing studies indicated that circRNAs play important roles in age-related disease. Atherosclerosis (AS) is a common age-related disease, and a research found that circPTPRA was upregulated in serum samples of AS patients, which promoted cell proliferation and inhibited cell apoptosis through repressing miR-636 and upregulating SP1 signaling axis (Zhang, 2020). In addition, in PPI network analysis, there were 11 proteins associated with the other five proteins; thus, they could be considered as hub proteins in this network. These proteins, such as Tgoln2, Prkca, and Hsp90aa1, are mostly structural proteins, and some enzymes that participate in the metabolism of macromolecules, which is in accordance with the results of GO analysis.

## Conclusion

In summary, this study showed the profiles of differentially expressed circRNAs and their mRNAs in age-associated MSC senescence. Our results suggest that circRNAs may play an important role in this process. Considering that only a small number of the total circRNA population has been studied, the investigation of circRNAs is underway. Therefore, additional studies are required to elucidate the significance of the change of circRNAs in the process of cellular senescence, which may contribute to the biological and molecular mechanisms and facilitate the development of novel therapeutic approaches.

## Data Availability Statement

The datasets presented in this study can be found in online repositories. The names of the repository/repositories and accession number(s) can be found below: SUB9796502, SRA: SRP322768, and BioProject: PRJNA735212.

## Ethics Statement

The animal study was reviewed and approved by the Experimental Animal Center of Jilin University, Changchun, China (permit number: SYXK 2018-0001).

## Author Contributions

XH contributed conception and design of the study. HS, YSu, XY, XG, HW, LZ, and YSh were responsible for finding literature and extracting data. HS conducted data analysis and wrote the first draft of the manuscript. XH contributed to manuscript revision and final approval for submission. All authors reviewed the manuscript and provided comments.

## Conflict of Interest

The authors declare that the research was conducted in the absence of any commercial or financial relationships that could be construed as a potential conflict of interest.

## Footnotes

1 https://string-db.org
